# Urinary metabolic phenotyping for Alzheimer’s disease

**DOI:** 10.1038/s41598-020-78031-9

**Published:** 2020-12-10

**Authors:** Natalja Kurbatova, Manik Garg, Luke Whiley, Elena Chekmeneva, Beatriz Jiménez, María Gómez-Romero, Jake Pearce, Torben Kimhofer, Ellie D’Hondt, Hilkka Soininen, Iwona Kłoszewska, Patrizia Mecocci, Magda Tsolaki, Bruno Vellas, Dag Aarsland, Alejo Nevado-Holgado, Benjamine Liu, Stuart Snowden, Petroula Proitsi, Nicholas J. Ashton, Abdul Hye, Cristina Legido-Quigley, Matthew R. Lewis, Jeremy K. Nicholson, Elaine Holmes, Alvis Brazma, Simon Lovestone

**Affiliations:** 1 European Molecular Biology Laboratory, European Bioinformatics Institute, EMBL-EBI, Wellcome Trust Genome Campus, Hinxton, CB10 1SD UK; 2 Department of Metabolism, Digestion and Reproduction, National Phenome Centre, Imperial College London, Hammersmith Campus, IRDB Building, London, UK; 3 UK Dementia Research Institute, Hammersmith Hospital, Imperial College London, London, W12 0NN UK; 4 Division of Systems Medicine, Imperial College London, South Kensington Campus, London, SW7 2AZ UK; 5 IMEC, Leuven, Belgium; 6 Department of Neurology, University of Eastern Finland and Kuopio University Hospital, Kuopio, Finland; 7 Medical University of Lodz, Lodz, Poland; 8 Institute of Gerontology and Geriatrics, University of Perugia, Perugia, Italy; 9 3rd Department of Neurology, Aristotle University, Thessaloniki, Greece; 10 INSERM U 558, University of Toulouse, Toulouse, France; 11 King’s College London, Institute of Psychiatry, Psychology and Neuroscience, London, UK; 12 Department of Psychiatry and Neurochemistry, Institute of Neuroscience and Physiology, The Sahlgrenska Academy, University of Gothenburg, Gothenburg, Sweden; 13 Wallenberg Centre for Molecular and Translational Medicine, University of Gothenburg, Gothenburg, Sweden; 14 NIHR Biomedical Research Centre for Mental Health and Biomedical Research Unit for Dementia at South London and Maudsley NHS Foundation, London, UK; 15 Department of Psychiatry, Warneford Hospital, University of Oxford, Oxford, UK; 16 Present Address: Health Futures Institute, Murdoch University, Perth, WA 6105 Australia; 17 Present Address: The Perron Institute for Neurological and Translational Science, Nedlands, WA 6009 Australia; 18 Present Address: Janssen-Cilag Ltd, High Wycombe, UK

**Keywords:** Biomarkers, Dementia, Alzheimer's disease, Metabolomics, Genomics

## Abstract

Finding early disease markers using non-invasive and widely available methods is essential to develop a successful therapy for Alzheimer’s Disease. Few studies to date have examined urine, the most readily available biofluid. Here we report the largest study to date using comprehensive metabolic phenotyping platforms (NMR spectroscopy and UHPLC-MS) to probe the urinary metabolome in-depth in people with Alzheimer’s Disease and Mild Cognitive Impairment. Feature reduction was performed using metabolomic Quantitative Trait Loci, resulting in the list of metabolites associated with the genetic variants. This approach helps accuracy in identification of disease states and provides a route to a plausible mechanistic link to pathological processes. Using these mQTLs we built a Random Forests model, which not only correctly discriminates between people with Alzheimer’s Disease and age-matched controls, but also between individuals with Mild Cognitive Impairment who were later diagnosed with Alzheimer’s Disease and those who were not. Further annotation of top-ranking metabolic features nominated by the trained model revealed the involvement of cholesterol-derived metabolites and small-molecules that were linked to Alzheimer’s pathology in previous studies.

## Introduction

Unmet medical need and the repeated failure of clinical trials in Alzheimer’s disease (AD) have together resulted in a surge of research seeking to understand disease mechanisms and generate novel therapeutic approaches. In order for such therapies to succeed it is widely accepted that trials will need to be performed early in the disease process^1,2^. Currently, the optimal biomarkers used to detect AD processes early in the course of the disease are Positron Emission Tomography (PET) imaging and cerebrospinal fluid (CSF) markers^3^. However, PET imaging is not universally available and obtaining CSF is a relatively invasive procedure. Progress has been made in the attempt to supplement these relatively specific biomarkers with other biomarkers that might be more applicable to larger populations using, for example, blood biomarkers. Putative markers in blood have been identified using proteomics, transcriptomics, metabolic and lipidomic phenotyping platforms. Efforts are now underway to replicate these and to generate single markers or biomarker panels that might be used as part of a process identifying people with early disease^4–6^.

There are relatively few studies examining the potential of urine as a biomarker fluid in AD^7–10^, probably because being separated from the brain not only by the blood–brain barrier but also by glomerular filtration, urine seems inherently unlikely to possess a signature of neurodegeneration. Most of the studies reported to date are small both in terms of the numbers of molecular targets and numbers of individuals examined. However, urine is a complex fluid with metabolites that reflect a response to injury^11^ and oxidative stress^12^ amongst other biological events at the systems level and might, therefore, be useful as a target fluid in neurodegeneration and other brain diseases^13^. An added advantage is that urine carries information on the metabolites arising from the gut microbiome^14^, an area of research that is gaining greater focus in neurodegeneration and AD^15,16^. In mouse models a range of methods to characterize the urine metabolome have been employed, with some success in identifying markers that differ between transgenic animals and controls^17–20^, but these models do not encompass the full systems effect and metabolic progression of AD in humans. Therefore deep exploration of the urinary metabolome for biomarkers relevant to AD may yield valuable mechanistic information. We report here the application of in-depth urinary metabolic phenotyping in a large multi-centre study consisting of four groups of participants. One group consisted of individuals clinically diagnosed with AD. A second group consisted of individuals with no apparent clinical symptoms of cognitive decline or dementia (control group—CTL). The final two groups consisted of individuals diagnosed with Mild Cognitive Impairment (MCI) who either remained cognitively stable throughout the follow-up term of the study (sMCI) or converted to a clinical AD diagnosis at a later study visit (cMCI). All samples that underwent metabolic phenotyping were collected at a single time point (baseline assessment).

Metabolic phenotyping was completed using a complementary dual-platform approach consisting of both proton nuclear magnetic resonance spectroscopy (^1^H-NMR) and ultra-high-performance liquid chromatography coupled with mass spectrometry (UHPLC-MS) to ensure comprehensive metabolite coverage. Critically, in order to address the challenge of rich metabolic datasets, where analytical variables far exceed the numbers of samples available to study, we applied a novel approach consisting of metabolomic dataset normalisation followed by a feature reduction process that only selected those metabolites which associated with a metabolic regulatory genetic element—a technique known as Quantitative Trait Loci for metabolites (mQTL). In this way, we identified a panel of metabolites that might accurately classify the participant groups. Of particular interest was the ability of the classification model to differentiate between baseline MCI participants, who later either remained cognitively stable (sMCI) or converted to clinical AD (cMCI) which heralds promise for non-invasive scoping for early biomarkers of AD.

The classification model was used to prioritise metabolites for annotation according to their importance to predict AD. The analysis of the annotated metabolites revealed multiple direct and a few indirect links to the etiopathological processes in AD.

## Results

We report here the largest study to date of metabolic phenotyping analysis of urine as a potential marker of AD. Metabolic phenotyping was performed on urine obtained from the AddNeuroMed/Dementia Case Registry (ANM/DCR) cohorts^21–23^ using two analytical platforms: UHPLC-MS (${\hbox {n}}=561$ samples) and ^1^H-NMR (${\hbox {n}}=575$ samples). The cohort of participants used in the analysis includes participants with normal cognition (control group—CTL), stable mild cognitive impairment (sMCI), mild cognitive impairment converting to dementia (cMCI) and participants with Alzheimer’s disease (AD) (Table 1). The number of available samples and metabolic features is summarised in Table 2. Using an innovative approach to the challenge of very high dimensionality data analysis, we first prioritise a set of metabolites using mQTL mapping, ensuring that features with a degree of genetic regulation were selected. From this reduced feature selection, we were then able to select metabolites that are associated with AD and were able to predict conversion to dementia from MCI.

**Table 1:** Overview of study participants.

	CTL	sMCI	cMCI	AD	Total
**Number of participants**	214	200	55	197	666
Age					
Mean$\,\pm$ sd	$76.1 \pm 5.1$	$76.7 \pm 5.5$	$77.9 \pm 7.9$	$76.6 \pm 5.8$	
**Sex**					
Male	103	99	20	101	323
Female	111	101	35	96	343
**MMSE score**					
Mean $\;\pm \;$ sd	$28.9 \pm 1.1$	$27.3 \pm 1.7$	$26.3 \pm 1.8$	$20.0 \pm 4.3$	
Metabolic UHPLC-MS data obtained	172	167	45	177	561
Metabolic NMR data obtained	174	173	46	182	575
Metabolic data obtained (UHPLC-MS and NMR)	132	140	36	162	470
Genetic and metabolic UHPLC-MS data available	119	80	24	122	345
Genetic and metabolic NMR data available	120	83	23	126	352
APOE genotype available	154	155	44	174	527
**APOE genotype**					
E3E3	84	84	13	70	251
E3E4	37	50	24	66	177
E2E3	22	10	4	9	45
E4E4	7	7	3	27	43
E2E4	3	4	0	2	9
E2E2	1	1	0	0	2

*CTL* normal cognition (control) participants, *sMCI* stable mild cognitive impairment, *cMCI* mild cognitive impairment converting to dementia, *AD* participants with Alzheimer’s disease, *MMSE* Mini-Mental State Examination.

### Dimensionality reduction of metabolic features

Given that the number of metabolic features found in the cohort samples was orders of magnitude higher than the number of samples (specifically 55,675 features), as the first step we developed a novel feature reduction method. We utilised the availability of genetic data, in particular, 12 million SNPs obtained in the previous study^24^ and looked for an association between these SNPs and the metabolic features, performing metabolic quantitative trait locus analysis. We hypothesized that an association between a metabolite and a disease state is more likely to be relevant to an etiopathological mechanism if this metabolite was also associated with a genetic variant previously linked to a relevant disease phenotype.

**Figure 1:**
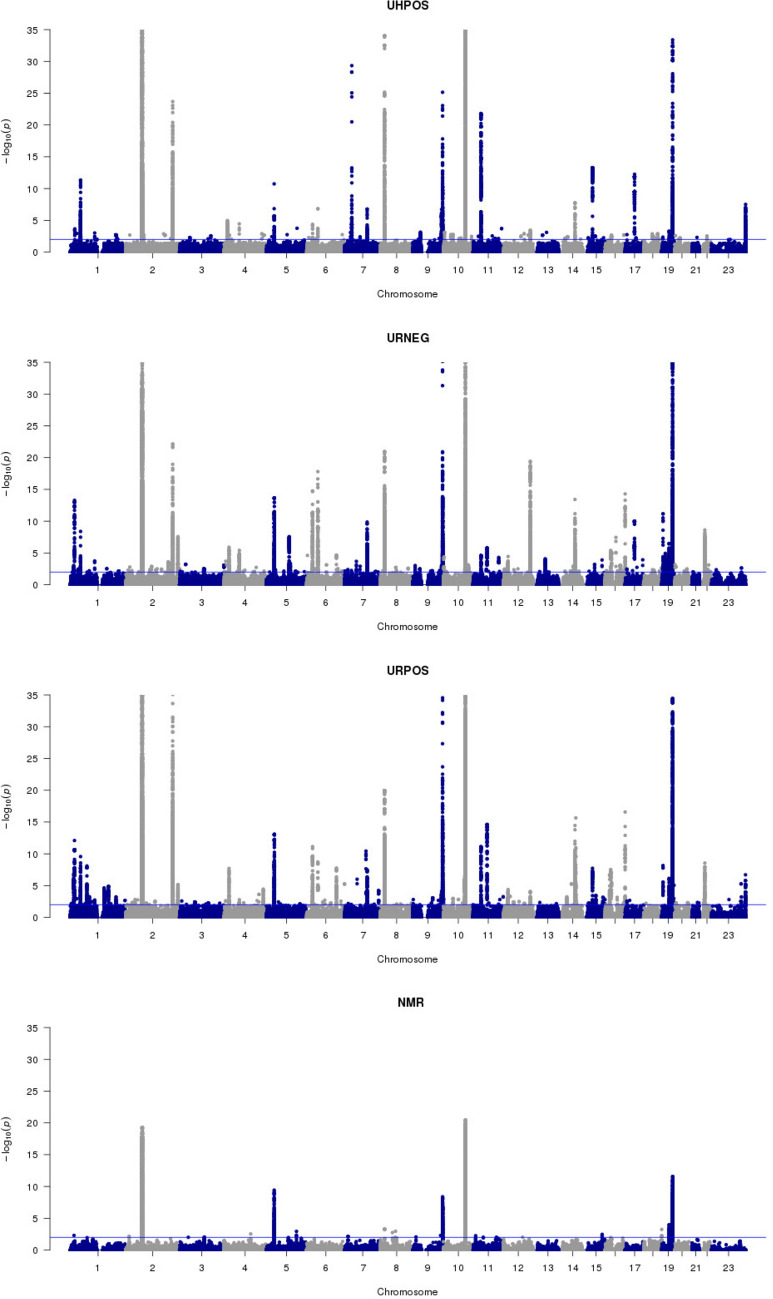
Manhattan plots presenting significant QTL associations with metabolic features across the metabolite phenotyping datasets. The x-axis shows each SNP that was analysed, sorted by chromosome and position. The y-axis shows the $-\;$log10 of the p-value for association with metabolic features concentration. Four sections correspond to four different metabolomic assays presented in our study: UHPOS, URNEG, URPOS and NMR.

The mQTL analysis resulted in a total of 1542 individual metabolic features relating to either a chemical shift in the case of ^1^H NMR (233 metabolic features) or a chromatographic retention time and mass to charge ratio (m/z) feature in the UHPLC-MS data (1309 features). The resultant metabolic features were associated with 6932 SNPs at a q-value $< 0.01$ (Table 3, Fig. 1). Of these, 6047 unique SNPs were linked to features from the UHPLC-MS data, 876 SNPs to features from the ^1^H NMR data, with 838 SNPs common to both. Given the 12 million SNPs tested, the probability of observing 838 or more SNPs in the intersection between UHPLC-MS and ^1^H NMR results by chance is vanishingly small (p-value < 2.23E−308). Previously, a total of 276 metabolomic QTLs have been reported^25–30^, and 83% of these SNPs were reproduced in the current study (Supplementary Materials Table S2), thereby, validating our pipeline.

**Table 2:** Summary of samples and metabolic features available for the analysis.

Platform	Assay	Abbreviation	# metabolic features	# samples	# metabolic QTL samples
UHPLC-MS	HILIC ESI+	UHPOS	6851	561	345
	RPC ESI−	URNEG	16,961	561	345
	RPC ESI+	URPOS	13,217	561	345
NMR	^1^H NMR	NMR	18,646	575	352

Details of used metabolomic platforms and assays are available in “Methods” section. Number of metabolic QTL samples—genetic and metabolic data availability.

**Table 3:** Metabolic QTL mapping results.

Assay	# SNP/metabolic feature associations	# unique SNPs	# unique metabolic features
UHPOS	26,256	3004	256
URNEG	50,251	3974	518
URPOS	46,617	4479	535
NMR	12,518	876	233

Numbers of associations between metabolic features and SNPs found using q-value cut-off 0.01, resulting in a number of unique metabolic features and a number of unique SNPs for each metabolomic assay.

**Table 4:** Performance of the final classification model.

Dataset	Balanced accuracy	AUROC	Sensitivity	Specificity	Positive predictive value	Negative predictive value
Final model AD vs CTL	0.9872	1	1	0.9744	0.9796	1
Final model cMCI vs sMCI	0.8037	0.8785	0.7778	0.8296	0.5490	0.9333

Performance of the final classification model in discriminating AD vs CTL and cMCI vs sMCI.

**Figure 2:**
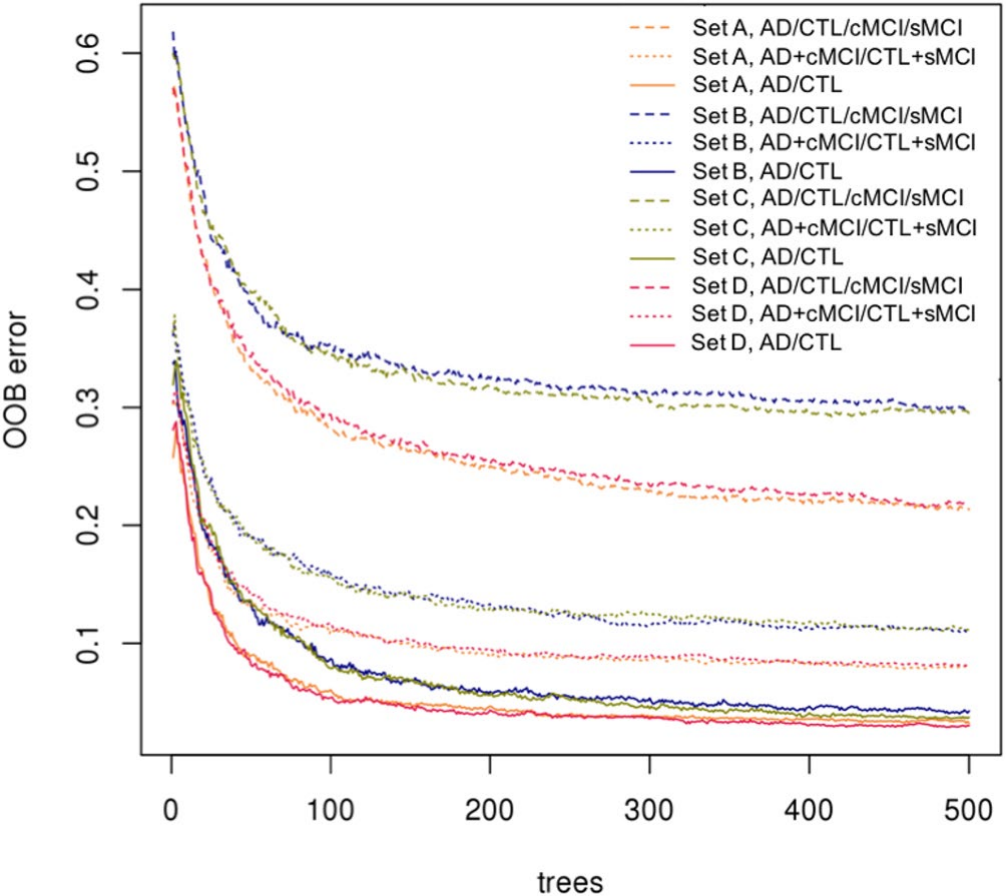
Performance of Random Forest models for different feature sets and three tested ways of classification. Tested feature sets: (**A**) metabolic features only, (**B**) metabolic and genomic features, (**C**) metabolic and genomic features together with sample covariates, and (**D**) metabolic features with sample covariates. Tested ways of classification: original multi-class—AD/CTL/cMCI/sMCI, binary over-sampling—AD + cMCI/CTL + sMCI, and binary under-sampling— AD/CTL. The best performing final model: set (**D**), binary under-sampling classification AD/CTL. The x-axis shows a number of trees used in the Random Forest run. The y-axis shows the Out-Of-Bug (OOB) prediction error.

### Ranking of metabolic features for annotation

Metabolite annotation remains the bottleneck and limitation of metabolic phenotyping studies^31,32^. After reducing the number of metabolic features to 1542, we aimed to prioritise them according to their relative importance of correctly predicting the AD state of the sample. We first built and tested Random Forests classification models on different combinations of features and diagnostic classes (for details see “Methods”). Briefly, we hypothesised that genomics data in combination with metabolic features and sample covariates could improve the prediction quality and tried combinations of following features: (1) 1542 metabolic features after the mQTL filtering, (2) 6932 SNPs associated with metabolic features and (3) sample covariates—age, sex and study site. Also, to account for the imbalance between the diagnostic classes, with the prevalence of AD and CTL classes over sMCI and cMCI classes, we applied re-sampling. First, we analysed all data in relation to the four original diagnostic categories (AD/CTL/cMCI/sMCI). Then, hypothesising that cMCI would be most similar to AD and sMCI most similar to control, we performed binary over-sampling by creating AD + cMCI and CTL + sMCI groups. Finally, we applied the under-sampling by using AD and CTL groups only. As the model trained on 1542 metabolic features together with sample covariates and only two diagnostic classes (AD and CTL) gave the lowest prediction error, it was selected as the final model (Fig. 2).

**Figure 3:**
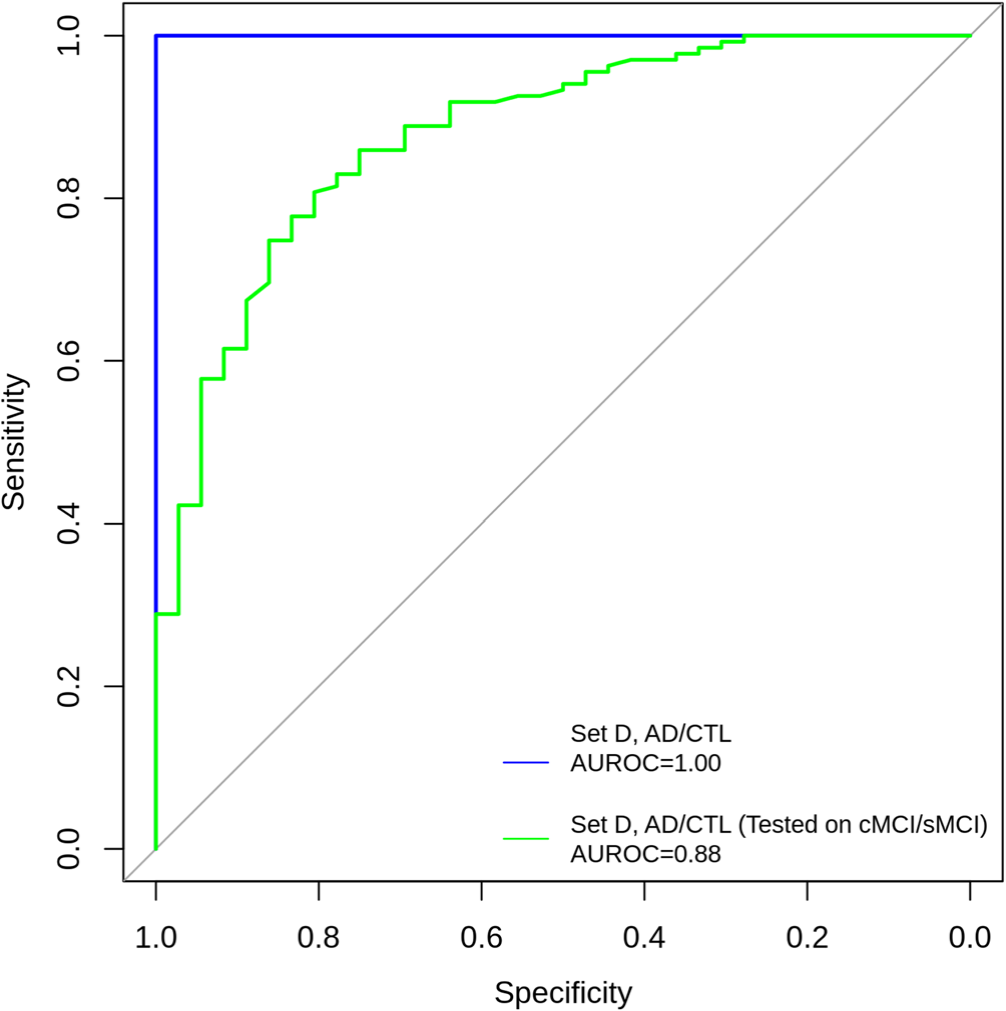
Receiver Operating Characteristic (ROC) curves for RF model discriminating AD vs CTL and then applied to cMCI and sMCI study groups. The model was trained with 1542 prioritised metabolic features and three covariates (age, sex, study site) identified from the AD vs CTL comparison only. The area under the ROC curve (AUROC) value for the AD vs CTL is 0.99. The AUROC value for cMCI vs sMCI classes is 0.88.

**Figure 4:**
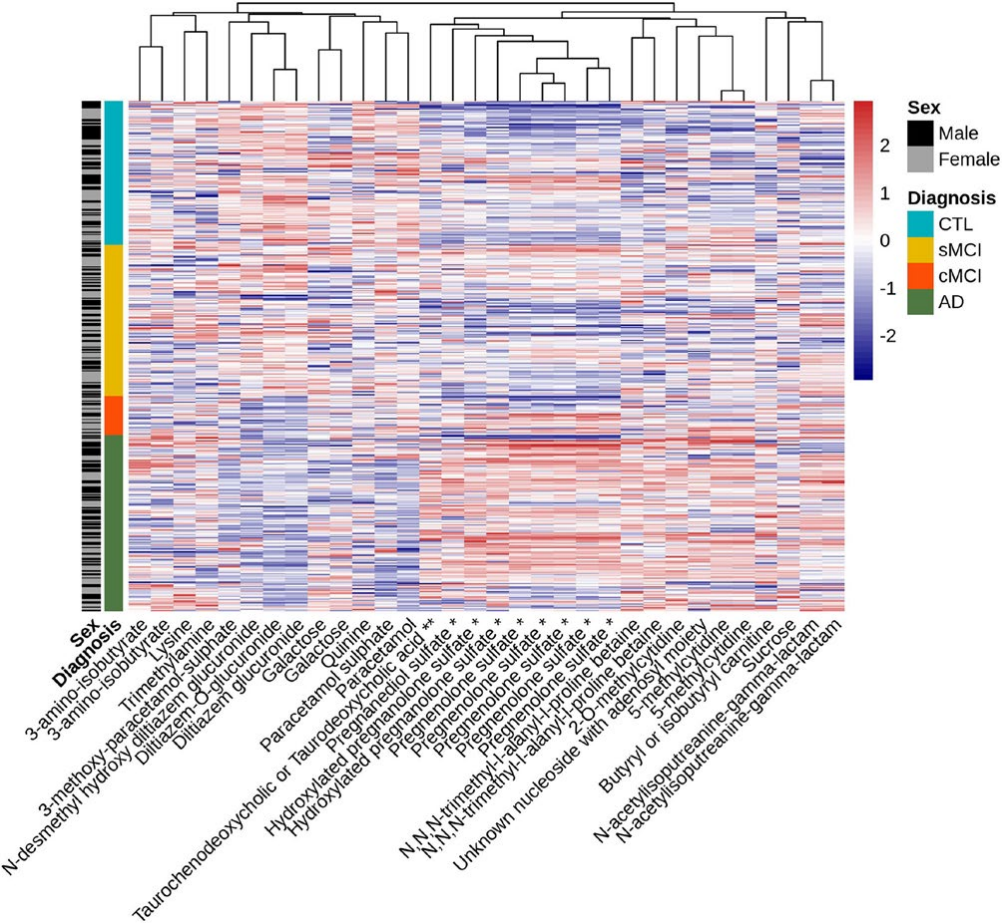
The heatmap showing concentrations of the annotated metabolic features. Note (*) indicates metabolite conjugation with N-acetylglucosamine, note (**) indicates metabolite conjugation with N-acetylglucosaminide. Columns sharing the same metabolite names are isomers of each other.

**Table 5:** Annotated metabolites.

Metabolite family	Metabolite annotation	Assay	#	PIC	F (3, 471)	p-value	CTL vs AD	CTL vs cMCI	CTL vs sMCI
Exogenous metabolites	N-Desmethyl O-desacetyl diltiazem glucuronide	UHPOS	1	0.04943	112.8	< 2E − 16	1.53 E − 11	1.54 E − 11	5.94 E − 02
	N-Desmethyl O-desacetyl hydroxy diltiazem glucuronide	UHPOS	1	0.02553	80.3	< 2 E − 16	1.53 E − 11	1.54 E − 11	3.32 E − 02
	N-Desmethyl hydroxy diltiazem glucuronide	URPOS	1	0.00741	40.13	< 2 E − 16	1.53 E − 11	1.54 E − 11	1.27 E − 01
	Paracetamol	UHPOS	1	0.02314	59.34	< 2 E − 16	1.53 E − 11	9.98 E − 01	7.30 E − 09
	Paracetamol sulphate	URPOS	1	0.003	26.66	6.19 E − 16	1.53 E − 11	9.26 E − 01	9.66 E − 01
	3-Methoxy-paracetamol sulphate	URPOS	1	0.00105	11.97	1.45 E − 07	4.00 E − 07	1.26 E − 01	7.85 E − 01
	Quinine	UHPOS	1	0.00028	13.26	2.56 E − 08	3.66 E − 07	2.66 E − 03	1.02 E − 06
Cholesterol derived metabolites	Hydroxylated pregnenolone sulphate N-Acetylglucosamine isomer 2*	URNEG	1	0.0051	34.75	< 2 E − 16	1.53 E − 11	5.61 E − 02	9.86 E − 01
	Hydroxylated pregnenolone sulphate N-acetylglucosamine isomer 1*	URNEG	1	0.01073	46.28	< 2 E − 16	1.53 E − 11	8.84 E − 03	8.26 E − 02
	Pregnenolone sulphate N-acetylglucosamine	URNEG	5	0.00463	26.42	8.39 E − 16	1.54 E − 11	4.87 E − 03	8.22 E − 02
	Pregnanediol sulphate N-acetylglucosamine	URNEG	1	0.00025	13.06	3.34 E − 08	3.02 E − 07	9.49 E − 01	9.30 E − 01
	Taurochenodeoxycholicc or taurodeoxycholic acid Nacetylglucosaminide*	URNEG	1	0.00052	20.31	2.18 E − 12	3.14 E − 10	9.99 E − 01	9.03 E − 01
Nucleosides, amines, carnitines, glycines	3-Aminoisobutyrate	NMR	2	0.00015	2.18	8.99 E − 02	9.44 E − 01	6.25 E − 01	3.75 E − 01
	N,N,N-Trimethyl-L-alanyl-L-proline betaine	URPOS	2	0.00299	34.09	< 2 E − 16	1.53 E − 11	8.65 E − 02	5.57 E − 02
	Butyryl or isobutyryl carnitine*	UHPOS	1	0.00054	14.54	4.57 E − 09	1.16 E − 08	7.25 E − 03	3.53 E − 01
	Trimethylamine	NMR	1	0.00014	1.68	1.71 E − 01	2.81 E − 01	9.48 E − 01	1.67 E − 01
	L-Lysine	NMR	1	0.00011	1.33	2.56 E − 01	9.90 E − 01	9.07 E − 01	4.65 E − 01
	5-Methylcytidine	UHPOS	2	0.00075	18.56	2.16 E − 11	2.67 E − 11	1.79 E − 01	9.89 E − 02
	2-O-Methylcytidine	UHPOS	1	0.00033	20.54	1.62 E − 12	1.93 E − 09	7.12 E − 01	9.79 E − 01
	Unknown nucleoside with adenosyl moiety	UHPOS	1	0.00026	9.24	5.99 E − 06	2.90 E − 06	1.71 E − 01	3.18 E − 01
	N-Acetylisoputreanine-gamma-lactam	URPOS	2	0.00046	13.42	2.06 E − 08	4.55 E − 09	1.74 E − 02	5.36 E − 03
Sugars	Sucrose	NMR	1	0.00027	0.17	9.20 E − 01	6.78 E − 04	5.99 E − 01	1.56 E − 01
	Galactose	NMR	2	0.0001	0.17	9.20 E − 01	9.05 E − 01	9.77 E − 01	9.74 E − 01

Note (*) signifies isomers that cannot be differentiated using mass spectrometry fragmentation data. Column headers: Assay—metabolomic assay; #—a number of metabolic features in the dataset; PIC—Permutation Importance Score from Random Forest algorithm showing the importance of metabolite for classification purpose; F (3, 471)—ANOVA results (MANOVA in case of multiple metabolic features) presented as F-statistic; p-value—ANOVA (MANOVA in case of multiple metabolic features) results presented as adjusted p-value; CTL vs AD, CTL vs cMCI and CTL vs sMCI—post hoc tests results presented as adjusted p-value. In the last four columns, Scientific Notation is used due to the presence of very small numbers.

**Table 6:** Annotated metabolites with mQTL results, phenotypic traits and literature findings.

Metabolic pathway	Metabolite annotation	Chr	Genes/genomic region	Phenotypic traits	Relationship to AD
	N-Desmethyl *O*-desacetyl diltiazem glucuronide				
	N-Desmethyl *O*-desacetyl hydroxy diltiazem glucuronide	1	Regulatory feature: ENSR00000006069	Anxiety and major depressive disorder, Obesity-related traits	
	N-Desmethyl hydroxy diltiazem glucuronide	15	MESP2	Coronary artery aneurysm in Kawasaki disease	
	Paracetamol				
	Paracetamol sulphate				
	3-Methoxy-paracetamol sulphate	4 AND 17	SORCS2 AND CNTROB	Biopolar disorder, Interleukin-10 levels	SORCS2 belongs to the Vps10 receptor family that has previously been linked to neurodegeneration and AD^34–35^, and is known to play functional roles in protein transport. In addition, the receptor family includes the SORL1 gene that encodes protein SorLA—a key protein in amyloid-beta precursor protein (APP) processing^36^.
	Quinine	2	AOX1	Late-onset Alzheimer’s disease	The mQTL association links aldehyde oxydase 1 (AOX1) gene and quinine. AOX1 gene has a previously reported GWAS trait “Late-onset Alzheimer’s disease”^37^.
Cholesterol metabolism (CM)	Hydroxylated pregnenolone sulphate *N*-acetylglucosamine isomer 2*	7	CHN2	Age at onset, Alzheimer’s disease, Obesity-related traits, Psychosis	Beta-chimaerin (CHN2) gene plays a role in neural development by regulating Rac1 activity^38^ and is known to be downregulated with age. Through Rac1 activation, gene CHN2 is linked with Alzheimer’s disease^39^.
	Hydroxylated pregnenolone sulphate *N*-acetylglucosamine isomer 1*				
	Pregnenolone sulphate *N*-acetylglucosamine				
	Pregnanediol sulphate *N*-acetylglucosamine				
	Tauro(cheno) deoxycholic acid *N*-acetylglucosaminide *	5	UGT3A1	Blood metabolite levels, Primary biliary cholangitis (PBC)	Neither the gene UGT3A1 nor the PBC has a known relationship to AD, although we note that a progressive cognitive impairment different to delirium is a feature of PBC, independently of liver pathology^40,41^. In animal models of biliary cirrhosis that has led to memory impairment, hippocampal pregnenolone sulphate infusion resulted in a memory-enhancing effect^42^.
CM, gut microbiota	3-Aminoisobutyrate	5	AGXT2	Metabolite levels, Asymmetrical dimethylarginine levels, Symmetrical dimethylarginine levels	
Gut microbiota	*N,N,N*-Trimethyl-l-alanyl-l-proline betaine	11 AND 21	Regulatory feature: ENSR00000961656 AND intergenic variant		
	Butyryl or isobutyryl carnitine *	15	intergenic variant		
	Trimethylamine	10	PYROXD2	General cognitive ability, Obesity-related traits, Metabolite levels	
	l-Lysine	19	SLC7A9	Estimated glomerular filtration rate, Creatinine levels	
DNA methylation	5-Methylcytidine	4	CC2D2, FBXL5, FAM200B, BST1	Parkinson’s disease, Blood protein levels, Cerebrospinal fluid biomarker levels	The FBXL5 gene is a critical component of iron metabolism^43^. It is associated with Parkinson’s disease (PD) in a region of chromosome 4. Iron dysregulation has long been associated with both PD and AD^44^.
	2-*O*-methylcytidine	9	NUP188, DOLK, PHYHD1, SH3GLB2	Body mass index	The PHYHD1 gene encodes 2-oxoglutarate oxygensase, an amyloid-beta interacting protein that has been shown to be dysregulated in both AD brain and in transgenic models with plaque pathology^45,46^.
	Unknown nucleoside with adenosyl moiety	12	Intergenic variant		The nearest gene to mQTL region is SYT1. It encodes protein synaptotagmin—a novel cerebrospinal fluid biomarker for Alzheimer’s disease^47^.
Polyamine metabolism	*N*- Acetylisoputreanine-gamma-lactam	2	Long intergenic non-protein coding RNA LINC01914		
CM, insulin resistance	Sucrose	8	Intergenic variant		
	Galactose	19	FUT2	Estimated glomerular filtration rate, Cholesterol levels	

Note (*) signifies isomers that cannot be differentiated using mass spectrometry fragmentation data. We present phenotypic traits previously associated with a genomic region of interest, and possible linkage of found genes to AD processes.

To further validate the performance of the final model, MCI samples were used. The model proved effective in separating MCI samples from individuals who subsequently converted to dementia (cMCI) from those who remained stable (sMCI), finding that 82.96% of cMCI samples were predicted as AD and 77.78% of sMCI samples as CTL (Fig. 3). This provided additional evidence that our feature reduction method resulted in a meaningful set of metabolites enabling the detection of early AD patients, along with the previous validation of 83% of associated SNPs in AD-related literature. The AUROC value of the model was 0.99 (Table 4 and Fig. 3), showing that the final model was quite robust.

**Figure 5:**
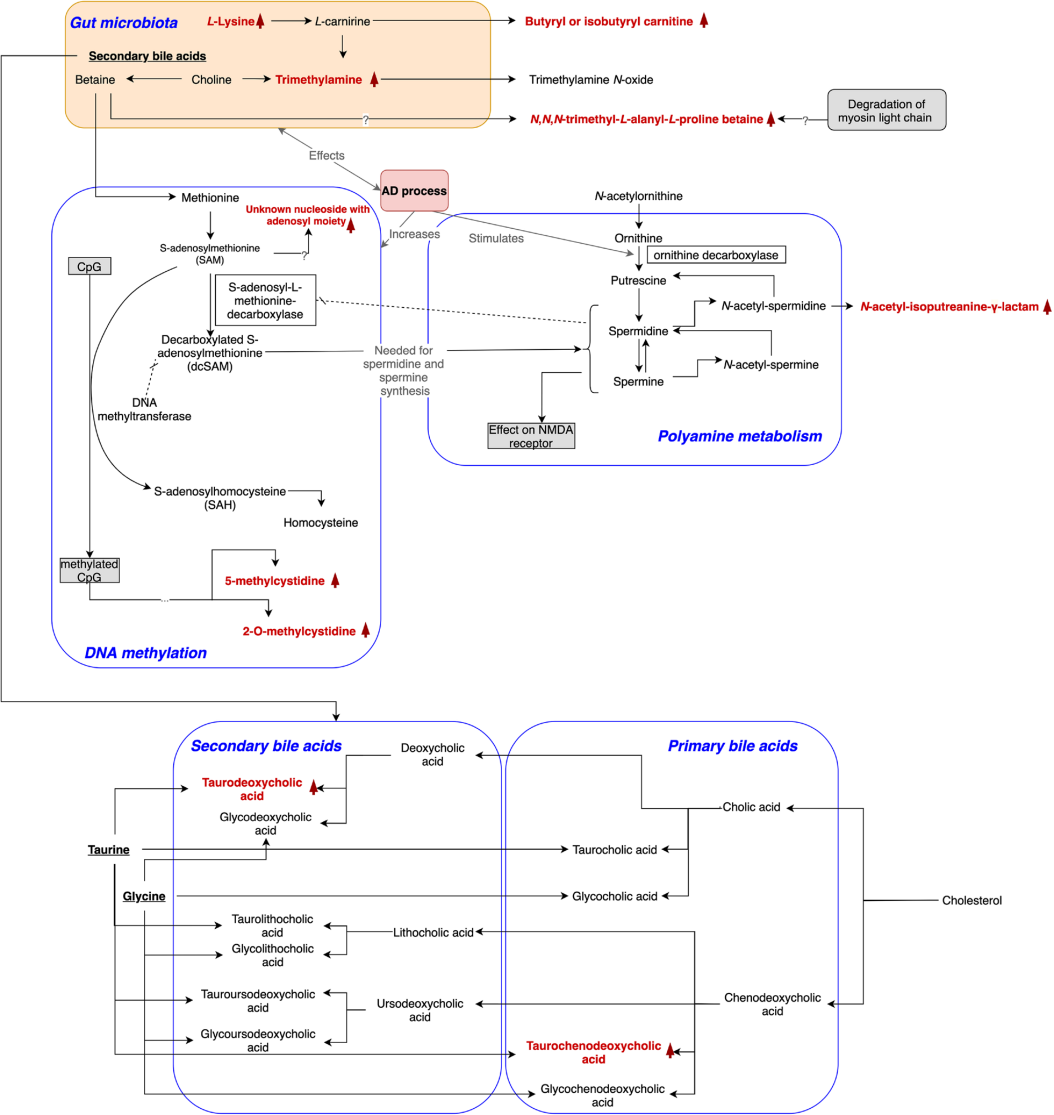
Annotated metabolites and their linkage to AD. Red colour indicates metabolites annotated in the study. Up arrow next to the metabolite’s name indicates increased levels in AD patients samples. Down arrow shows decreased levels in AD patients samples.

Next, we used the developed Random Forest model for prioritisation of the metabolic features by computing the permutation importance score for each metabolic feature (Supplementary Materials Table S3). This method gave us 235 metabolic features with a score of at least $10{^{-4}}$. Out of these, 32 features were successfully annotated by spectral interpretation. Since multiple analytical signals can correspond to the same metabolite, the annotation process resulted in a total list of 23 metabolites (Table 5).

### Analysis of annotated metabolites

We found three broad groups of annotated metabolites differentially present in the disease state: (1) conjugated metabolites of cholesterol derived compounds, (2) small molecule metabolites and (3) metabolites of exogenous sources.

Following the annotation, we quantified the differences in the levels of assigned metabolites across study groups using ANOVA (Table 5). A heatmap demonstrating inter-annotation correlations is shown in Fig. 4.

Having assigned chemical identity, we then investigated whether the associated SNPs were linked to any previously reported GWAS traits to gain potential insight into the relationship between metabolomic and genomic variations. We mapped any polymorphisms associated with our annotated metabolites to the GWAS Catalog’s SNPs^48^ by genomic regions ans summarised them in Table 6.

We speculate that all endogenous annotated metabolites are linked to AD processes through alteration of DNA methylation, gut microbiota malfunction and possibly altered metabolism of polyamines, cholesterol and sugar (Table 6, Fig. 5). The key observations from these analyses are reported in the sections below.

#### Cholesterol metabolism

There are three unique metabolites derived from cholesterol metabolism, all in the conjugated form: tauro(cheno)deoxycholic acid, pregnenolone and pregnanediol. These metabolites have higher levels in the AD and cMCI study groups (Table 5).

We found the two hormones, pregnenolone and pregnanediol, conjugated with sulfate and N-acetylglucosamine, both biotransformations were observed in human urine previously^49^.

Annotated tauro(cheno)deoxycholic bile acid (an isomer of taurochenodeoxycholic or taurodeoxycholic acid) is conjugated with an N-acetylglucosamine, known biotransformation prior to bile acid renal excretion^50^. There is an increased level of taurochenodeoxycholic or taurodeoxycholic acid conjugates in AD patients (see “Discussion” section).

#### Sugar metabolism and gut microbiota

We identified sucrose and galactose sugars with sucrose levels significantly higher in AD study group.

The following annotated metabolites are produced in or derived from gut microbiota processes: *N,N,N*-trimethyl-l-alanyl-l-proline betaine, 3-aminoisobutyrate, trimethylamine, lysine, butyrylcarnitine, and mentioned above, taurodeoxycholic acid. These metabolites have increased levels in AD patients (Table 5).

#### DNA methylation and polyamine metabolism

Evidence of DNA methylation in AD patients’ urine is found in the observation of two methylcytidine metabolites: 5-methylcytidine, and 2-*O*-methylcytidine. Another metabolite that shared the pattern of alterations with two methylated cytidine metabolites was annotated as “unknown nucleoside with adenosyl moiety”. We were not able to identify its structure definitively in this analysis but the mass spectrometric data provided the molecular formula C10H11N5O3, and fragmentation data indicated the presence of adenosyl moiety in the molecule (some details regarding structure elucidation effort are presented in the “Supplementary Document” “Metabolite_Annotation_details.docx”).

Another annotated metabolite is *N*-acetylisoputreanine-gamma-lactam, a catabolic product of spermidine. This metabolite levels alter not only in AD but also in cMCI and sMCI groups (Table 5).

#### Exogenous metabolites

Amongst exogenous metabolites, we found two prescribed medications, paracetamol and the calcium channel blocker diltiazem, in addition to the metabolite quinine. All three exogenous metabolites have associations with genetic variants (Table 6) and their levels significantly alter in study groups, as shown in Table 5.

## Discussion

Previous studies have reported data suggesting that a panel of metabolites circulating in blood was able to predict incipient AD with very high degrees of accuracy^51,52^, raising considerable hopes for finding pre-clinical AD biomarkers in blood. Subsequent studies using a similar, if not identical approach, in multiple, larger cohorts failed to replicate these findings^53–55^. Other studies have reported metabolic and lipidomic differences in blood from people with disease compared to age-matched controls with various degrees of power, success and outcome^56–59^. However, none of these studies have been unequivocally replicated. One of the limitations of studies with high dimensionality datasets and relatively small numbers of samples is susceptibility to over-fitting. Additionally, because of the inherent problems of heterogeneity in neurodegenerative disease and the diversity of analytical platforms used in metabolic profiling (^1^H-NMR, GC-MS, UHPLC-MS, CE-MS etc.), it is perhaps not surprising that there has been relatively little replication of metabolic phenotyping studies that seek biomarkers of disease. Similar problems plagued early genetic studies seeking susceptibility factors, but these have been largely overcome by the introduction of studies based on tens of thousands of individuals. In genetics it was possible to combine data from different cohorts using imputation techniques. This approach is more challenging for metabolic phenotyping approaches, which often utilize heterogeneous technologies and independent assays, the results from which are more difficult to build a comprehensive picture of the metabolic landscape. With increased throughput and lower cost of metabolic assays, such larger studies will become possible in future.

In the absence of studies with large sample size, one approach to combat the limitation of high dimensionality in molecular studies is to reduce the dimensionality of the data. To achieve this, here we used the mQTL approach. In doing so, we provide for the possibility of a degree of validation in other, much larger dataset derived from genetic associations studies, also enabling the inference of a degree of causality when an association is discovered. While this work requires replication, this finding holds promise for biomarkers in urine—arguably the most readily available biomarker fluid. Using this mQTL targeting approach, we show a highly significant association of a relatively small set of 32 metabolic features with AD. A model generated from these features not only accurately predicts the disease state, but more importantly, the same model when applied to samples from participants with the clinical diagnosis of mild cognitive impairment (MCI), distinguishes those subjects that subsequently progress to dementia (cMCI) from those that remain stable (sMCI). Note that, MCI is commonly referred to as a prodromal condition, but in fact, is a highly heterogeneous state defined by impaired cognitive function relative to age-adjusted norms. An easy to perform test to identify people in the prodromal phase of AD, distinguishing the subjects with MCI that are likely to convert to AD from those remaining stable, would be an important advance for the field. Our results suggest that the analysis of urinary metabolites might provide such a test.

Given that the sample size in our study is small, in the absence of a larger replication cohort, it is important to provide as much additional evidence as possible showing that the method we developed is reliable. One such type of evidence comes from the analysis of the annotated metabolites. One set of metabolites that we found is related to cholesterol—a critical biological precursor, required for the biosynthesis of downstream metabolites such as bile acids, hormones and steroids, which are commonly found in their conjugated form in the urine. Hormones and related steroidal structures derived from cholesterol are known to have a role in brain function. Observed in this study pregnenolone sulfate is a known neuro-steroid species^60,61^ reported to influence cognition in rodent models^62^ and patient studies, perhaps through its role in modulating gamma-aminobutyric acid subunit A (GABAA) and N-methyl d-aspartate (NMDA) receptors^61^. The other hormone we annotated—pregnanediol, a metabolite of pregnenolone, was previously reported to be lower in the urine of older men^62^, however, this has not been linked to AD. Concentrations of non-conjugated bile acids were observed to be altered in AD in human blood and brain samples and in transgenic models of the disease^58,63^. A recent study on mouse models discovered that bile acids strongly inhibit the cysteine dioxygenase type-1-mediated (CDO1-mediated) cysteine catabolic pathway resulting in depletion of the free cysteine pool and reduction of the glutathione concentration^64^. Here, we found an increased level of taurochenodeoxycholic or taurodeoxycholic acid conjugates in AD patients. Taurodeoxycholic acid, is the secondary bile acid that was previously observed to be increased in AD patients as the result of hypothesised gut microbiota malfunction^65^. The need for further investigation of cholesterol related metabolites in AD pathology is strongly supported both by previous research and by our study results.

Sugar metabolism was previously implicated in AD pathophysiology linking dysregulation in glucose metabolism and insulin resistance^56^. We found sucrose and galactose sugars to be important for the AD classification. Studies in mice suggest that sucrose disrupts mitochondrial activity and promotes amyloid deposition in the brains of transgenic AD mice^66^. Additionally, treatment of ovariectomised rats using d-galactose leads to AD-like pathology and the development of AD in a rodent model^67^. The researchers reported the observed AD pathology was prevented following injections of 17-$\upbeta$ estradiol, suggesting a potential interlinked role for disruptions in sugar and sex hormone metabolism, alternations of both are reported in our results. However, it is necessary to highlight that we have very limited known covariates of this retrospective data cohort that was collected ten years ago. Original study participants exclusion criteria included other neurological or psychiatric diseases, significant unstable systemic illness or organ failure and alcohol or substance misuse^21^. That knowledge gives us some assurance that study participants did not have other diagnosed medical conditions like diabetes mellitus type 2 and renal disease.

Evidence of gut microbiota malfunction is a noticeable trait in our findings. We annotated trimethylamine, *N,N,N*-trimethyl-l-alanyl-l-proline betaine, 3-aminoisobutyrate, l-lysine and butyrylcarnitine metabolites with different levels of concentrations amongst study groups. We detected several metabolites closely linked to betaine, though not betaine directly. Betaine is a source for trimethylamine production and an alternate methyl source for converting homocysteine to methionine^68^, increasing DNA methylation and altering gene expression^69^. We detected trimethylamine, a metabolite released in gut microbiota from trimethylamine-containing dietary phospholipid components such as choline, lecithin, l-carnitine and mentioned above betaine. The oxidation of trimethylamine generates trimethylamine *N*-oxide that was reported to be elevated in Alzheimer’s patients^15^. In addition, observed here *N,N,N*-trimethyl-l-alanyl-l-proline betaine is a recently discovered plasma biomarker of kidney function. Plausibly this metabolite is a product of betaine and myosin light chain degradation^70^, though this hypothesis has yet to be confirmed. Another detected product of gut microbiota processes is butyrylcarnitine—a product of l-carnitine processing in the human body. Previous research conducted in the mouse brain has shown that in old age, the AD genetic load significantly increase levels of butyrylcarnitine^71^. Changes in butyrylcarnitine concentrations together with the observation of elevated levels of lysine in AD patients suggest alterations of carnitine in AD, since lysine is one of the sources for carnitine production in humans. These complex metabolic processes in gut microbiota require further investigation.

We speculate that changes in DNA methylation during AD processes are verified through observed alterations of 5-methylcytidine and 2-*O*-methylcytidine. These results correlate with recent reports showing significant alterations in 5-methylcytidine in early stages of Alzheimer’s disease^72^. Alterations in the concentrations of *N*-acetylisoputreanine-gamma-lactam, a catabolic product of spermidine that is formed from *N*-actetylspermidine^73^ support a potentially altered metabolism of polyamines in AD: the stimulation of ornithine decarboxylase in the AD process leads to increased levels of *N*-acetylspermidine and spermidine^74^.

Exogenous metabolites found in this study and their linkage with genomic variants (paracetamol and quinine) or with possible dementia protective effect (diltiazem metabolomic profile) are significant findings for further investigation. Given that the prescription of quinine preparations is recommended only for malaria prophylaxis, this is an unlikely explanation for the quinine finding in this European population of older people. However, its prevalence in dietary sources may explain its presence in the population. This finding, along with the reporting of altered levels of diltiazem and paracetamol in AD is not obviously explicable, although reverse causality, where the disease state is resulting in a change in either prescription or compliance with medication, is an obvious possible explanation of the finding.

While the strengths of the study lie in the mQTL approach and the acquisition of a large amount of metabolic phenotyping data, there are undoubtedly limitations to consider. First, although large compared to previously reported urine studies, the analysis remains vulnerable to over-fitting and bias as the number of features is three times that of the number of samples, even following the mQTL based feature reduction. Clearly, larger and independent datasets are necessary to replicate the findings we report here. This could be achieved using a targeted quantitative mass spectrometry assay, specifically designed to quantitate the metabolic pathways of the key metabolites identified here. This would both help validate the findings and provide greater insight into pathway mechanisms and the networks involved. Secondly, the cohort used here, lacks specific diagnostic biomarkers (such as PET or CSF measures) indicative of pathological load and the diagnostic categorisation rests on experienced clinician assessment together with systematic assessment by a research worker, albeit using a widely tested and proven methodology. Ideally, data would also be collected on diet and life-style factors such as exercise and other environmental exposures together with detailed assessments of co-morbid and prior medical history and medication use. Such exploration of factors beyond diagnostic category that might influence the metabolic profile will be important topics for future studies. Lastly, by focusing on the metabolites with an associated genetic variant, we may exclude metabolites that have a significant association with AD but are largely influenced by the environment, lifestyle and not as strongly by genetics. The further research of exogenous metabolites, such as medicines and dietary compounds, as well as effects of environmental factors on metabolic processes in AD is necessary.

## Methods

We confirm that: all experiments were performed in accordance with relevant guidelines and regulations; all methods were carried out in accordance with relevant guidelines and regulations; all experimental protocols were approved by the European Union AddNeuroMed program.

### Study participants

The data and biospecimens were collected (AddNeuroMed/Dementia Case Registry (ANM/DCR) cohort) in European, multi-site study, public-private partnership^21–23^. The cohort includes participants with established AD, MCI and normal cognition who were evaluated using well-established systematic interviews for diagnosis. Formally, the diagnoses are not pathologically confirmed or supported by specific biomarkers of pathology, but it has been shown that this diagnostic method is highly predictive using MRI scans^21^ and subsequent post-mortem classification^75^. In addition, all disease categories, and conversion to dementia from MCI, were diagnosed by an experienced clinician according to criteria as described below. Briefly, the inclusion and exclusion criteria for study groups were as follows. Inclusion criteria for AD study group: (1) ADRDA/NINCDS and DSM-IV criteria for probable Alzheimer’s disease; (2) mini Mental State Examination score ranged from 12 to 28; (3) age 65 years or above. Inclusion criteria for MCI and CTL study groups: (1) mini Mental State Examination score range between 24 and 30; (2) geriatric Depression Scale score less than or equal to 5; (3) age 65 years or above; (4) medication stable; (5) good general health. The distinction between MCI and controls was based on two criteria: (1) subject scores 0 on Clinical Dementia Rating Scale = control; (2) Subject scores 0.5 on Clinical Dementia Rating scale = MCI. For the MCI subjects it was preferable that the subject and informant reported occurrence of memory problems. All AD subjects had a CDR score of 0.5 or above. The distinction between cMCI (converted MCI) and sMCI (stable MCI): based on follow-up interviews and tests. Exclusion criteria (all study groups): (1) significant neurological or psychiatric illness other than AD; (2) significant unstable systematic illness or organ failure; (3) alcohol or substance misuse.

All samples were collected under human participant research protocols, including informed consent containing the clause necessary for allowing bio-specimens and data sharing abroad. Blood and urine were collected at baseline and stored at $-80^{\circ }{\hbox {C}}$. Genetic data from blood samples were obtained using the Illumina 610-Quad chip in two different batches as previously described^24^, while the third batch was obtained using HumanOmniExpress 24 v1.1. For this study morning (non-first void) urine samples were collected at baseline assessment and were subjected to metabolic profiling analysis via UHLC-MS and by ^1^H NMR spectroscopy (561 and 575 samples respectively) (Table 1).

### ^1^H NMR metabolic phenotyping

Samples were analyzed by ^1^H-NMR spectroscopy in-line with previously published standard protocols for the study of human urine samples^76^. In brief, samples were prepared using a Gilson 215 liquid handling robot and transferred to 4 in. length $\times$ 5 mm outer diameter NMR tubes in batches of 80 patient samples and four QC pooled samples. Racks of 96 prepared NMR tubes were transferred to a refrigerated SampleJet sample handler robot (Bruker Co) working at $5^{\circ }\hbox {C}$. One dimensional ^1^H-NMR general profile and two-dimensional J-resolved (Jres) experiments were acquired on a Bruker Avance III HD 600 spectrometer following the set up previously described^76^. Experiments were acquired and processed in automation using TopSpin 3.2 and ICON NMR. Phasing, baseline correction and calibration to TSP were also carried out in automation after each acquisition. Spectra quality was assessed using an in-house developed bioinformatics tool nPYc^77^ following the quality criteria previously described^76^.

### UHPLC-MS metabolic phenotyping

UHPLC-MS analysis of urine samples was performed as previously described^78^ utilizing a combination of reversed-phase chromatography (RPC) and hydrophilic interaction chromatography (HILIC). UHPLC was performed using Waters Acquity UPLC systems (Waters Corp., Milford, MA, USA), coupled to Waters Xevo G2-S QTOF mass spectrometers (Waters Corp., Wilmslow, UK) via Z-spray electrospray ionization (ESI) sources. RPC separations were paired with both positive and negative ion mode detection (generating URPOS and URNEG datasets respectively), while the HILIC separation was paired with positive ion mode detection only (UHPOS). All UHPLC-MS datasets underwent feature extraction using Progenesis QI 2.1 software (Nonlinear Dynamics, Newcastle, UK) as previously described, and feature filtering was performed using previously described quality control materials^78^. Preprocessing including batch and run order correction was performed using an in-house developed bioinformatics tool nPYc^77^. Features were removed from the data sets where their analytical variation, assessed by repeated measurement of a pooled quality control sample (study reference), exceeded 30% (relative standard deviation) or where the analytical variation exceeded the total observed variation among all study samples. Features were also inspected for correlation between their observed intensity and sample dilution within a pooled QC dilution series and features with Pearson correlation coefficients less than 0.7 were removed.

### Metabolic QTL mapping

The goal of QTL mapping is to identify associations between genetic markers and phenotypic variation^79^, in the case of metabolic QTL, the genome-wide contribution of individual alleles to a metabolic feature concentration^27^. The R package MatrixEQTL^80^ was used for QTL mapping by modelling the effect of genotype as additive linear including covariates to account for: age, sex, data collection centre and cohort. The null-hypothesis, “there is no QTL effect for the metabolic feature concentration”, was tested against the alternative, “there is QTL effect between SNP and metabolic feature concentration”, for each pair of metabolic feature and SNP. We corrected results for multiple comparisons by calculating q-value using the Benjamini–Hochberg procedure^81^. The number of metabolic features and SNPs found to be associated using stringent q-value threshold 0.01 is presented in Table 3.

#### Data preparation For mQTL analysis

UHPLC-MS metabolic datasets UHPOS, URPOS and URNEG were normalised with EigenMS method^82^ that removes bias of unknown complexity from this type of metabolomics experiment at the same time preserving known bias, diagnoses in the case of this study. Subsequently, both EigenMS processed UHPLC-MS data, and raw one-dimensional ^1^H NMR metabolic data were normalised using the quantile normalisation^83^ method to make metabolomics data suitable for the QTL mapping.

#### Imputation and quality control of genomics data

After quality control using PLINK^84^, genomics data from separate batches were assembled and remapped to “hg19-build37” reference genome. The imputation was performed with IMPUTE2 software^85^. Since SNPs with low minor allele frequency are non-informative and have a potential of creating spurious findings, the imputation results were converted into matrix form and filtered using minor allele frequency threshold 10%. The final genomics matrix includes 12,105,785 SNPs. The results of population stratification indicated biases by cohort and data collection centres (Supplementary materials Figures S1 and S2).

### Model selection

We used the Random Forests algorithm^86^ to prioritise metabolic features that can potentially be used as Alzheimer’s disease biomarkers. The main focus of this study is on metabolic features. However, there are also 6923 SNPs that metabolic features are associated with and covariates available for the samples: age, gender and data collection centre. To find the best prediction model, we considered the following sets of features:

A. Metabolic features only;
B. Metabolic features and SNPs;
C. Metabolic features, SNPs and covariates;
D. Metabolic features and covariates.

As discussed earlier, we tested three different ways of classifying diagnostic groups: four original diagnostic categories (AD/CTL/cMCI/sMCI), binary over-sampling AD + cMCI/CTL + sMCI, and binary under-sampling AD/CTL by removing cMCI and sMCI data. We explored different classifications approaches and feature sets by using the R implementation of RF^87,88^ to find the best combination. As the comparison criteria, we used the Out-Of-Bag (OOB) errors which is a standard approach for Random Forests. OOB error is a method of measuring the prediction error utilising bootstrap aggregation to subsample data used for training. It helps to avoid the need for an independent validation dataset^89^. Results in the form of OOB errors of the RF models built for the different combinations of feature set and diagnostic groups are shown in Fig. 2. Each model was repeated ten times, using an increasing number of trees per run. The binary classification AD/CTL gives the best OOB errors for all four feature sets: the feature set A—0.0325, B—0.0404, C—0.0363 and D—0.0292, correspondingly. The feature set D, metabolic features and covariates, and binary classification AD/CTL, Alzheimer’s disease and healthy control samples only, after the tuning of RF parameters, gave the OOB predicted error 0.0241. The value of the area under the receiver operating characteristic curve (AUROC)^90^ for the model is 0.99 (Table 4 and Fig. 3). This model is our final RF model used in further analysis.

#### Tuning of random forests parameters

There are two parameters to tune for the RF algorithm: the number of trees used in the forest—ntree, and the number of variables used in each tree-mtry. We applied the alternating iterative procedure to find the best possible parameters values for ntree and mtry. The results plotted on Supplementary materials Figure S3 shows that with the ntree value equal to 680 the out-of-bag error rate stabilises and reaches its minimum 0.0254 with standard deviation 0.0056. The best mtry value we found is 90 (Supplementary materials Figure S4). It gives minimal OOB error rate 0.0241 with standard deviation equals to 0.0046.

### Ranking of metabolic features

We ranked metabolic features using the permutation importance score obtained from the final RF model. This score is based on the idea that if the feature is not essential, then rearranging the values of that variable does not degrade classification accuracy. The list of ranked metabolic features with calculated permutation importance score and added metabolite annotations are presented in the Supplementary materials Table S3.

### Metabolite annotation

For metabolite identification features of interest derived from the UHPLC-MS datasets first underwent correlation analysis using an R script developed in-house. This enabled the observation of co-eluting adducts and in-source fragments that are characteristic of metabolites. Further structural data were obtained via the use of high-resolution accurate mass to charge ratio (m/z) values and collision-induced dissociation (CID) fragmentation patterns. CID experiments were completed at a range of collision voltages at 5 V step intervals (5–45 V). The front quadrupole of the QTOF MS system was engaged to select for the feature of interest prior to CID. Chromatographic retention time matching to in-house standards was also completed where the standard was available for purchase. Online databases such as METLIN^91^, and the Human Metabolome Database (HMDB)^92^ were also used to assist with metabolite identification. Where available, analytical standards were purchased and spiked into representative samples to increase confidence in the annotation.

Tentative annotation of features of interest derived from ^1^H NMR analysis was completed by searching on literature and using in-house databases to match possible patterns of interest with the relevant spectra of standard compounds. The multiplicity of the signals of interest was confirmed using the corresponding Jres spectrum and a cassette of 2D spectra of representative samples including ^1^H,^1^H-COSY, ^1^H,^1^H-TOCSY, ^1^H,^13^C-HSQC.

## Supplementary information

Supplementary Information.

## Data Availability

Genomics data in European Nucleotide Archive PRJNA266531.

## Code availability

Github repository.
