# Supplementary material for: Urinary metabolic phenotyping for Alzheimer’s disease
Source: Sci Rep. 2020 Dec 10;10:21745. doi: 10.1038/s41598-020-78031-9 (PMC7730184; doi:10.1038/s41598-020-78031-9)
Supplement: Supplementary file 1 — Supplementary Information. [file 41598_2020_78031_MOESM1_ESM.zip › SupplementaryData/Metabolite_Annotation_details.docx]

**Urinary metabolic phenotyping for Alzheimer’s disease**

## Supplementary Document describing metabolite annotation

## Diltiazem metabolites

The main Phase I metabolites of diltiazem in humans are O-desacetyl diltiazem, N-desmethyl diltiazem, and N-desmethyl-O-desacethyl diltiazem (Koba, O. et al., 2016). The structure modifications during this stage of metabolism lead to the formation of functional groups (e.g. OH) that can be further metabolised (in the Phase 2) to glucuronides and/or sulphates.

In this work, we detected and annotated three metabolites of diltiazem. Two features were detected in the LC-MS HILIC positive assay UHPOS 5.01_535.1753m/z and 5.51_551.1705m/z, both corresponding to [M+H]^+^ ions (Table 5 and Table S4), and one feature was observed in the LC-MS RPC positive assays URPOS 4.64_637.1565m/z corresponding to [M+2Na-H]^+^ ion (Table 5 and Table S4) with the [M+H]^+^ at m/z 593.18.

MS/MS analysis (Figure S5) revealed that all three of them were glucuronide conjugates of different Phase I metabolites of diltiazem shown in Figure S6. Note that Metabolite 1 and Metabolite 3 can be represented by various isomers (depending on the position of the hydroxy group).


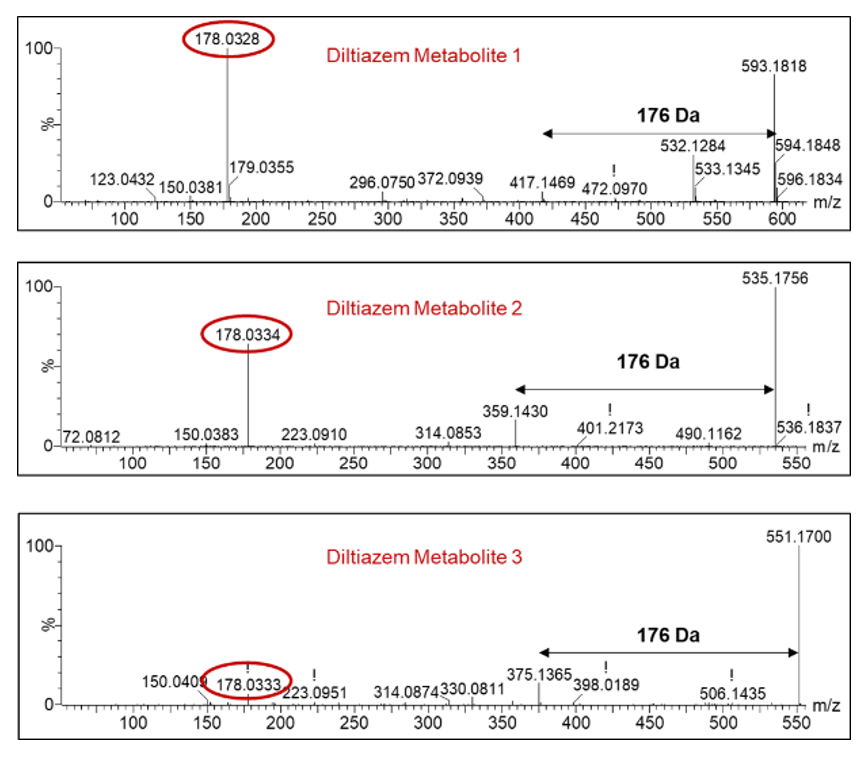


**Figure S5.** **MS/MS spectra of the three diltiazem metabolites**. Metabolite 1 was detected in RPC positive assay (URPOS) while Metabolites 2 and 3 were detected in HILIC positive assay (UHPOS). All three metabolites show loss of 176 Da characteristic of glucuronide conjugates and show a fragment at m/z 178.03 corresponding to the diltiazem fragmentation pattern.


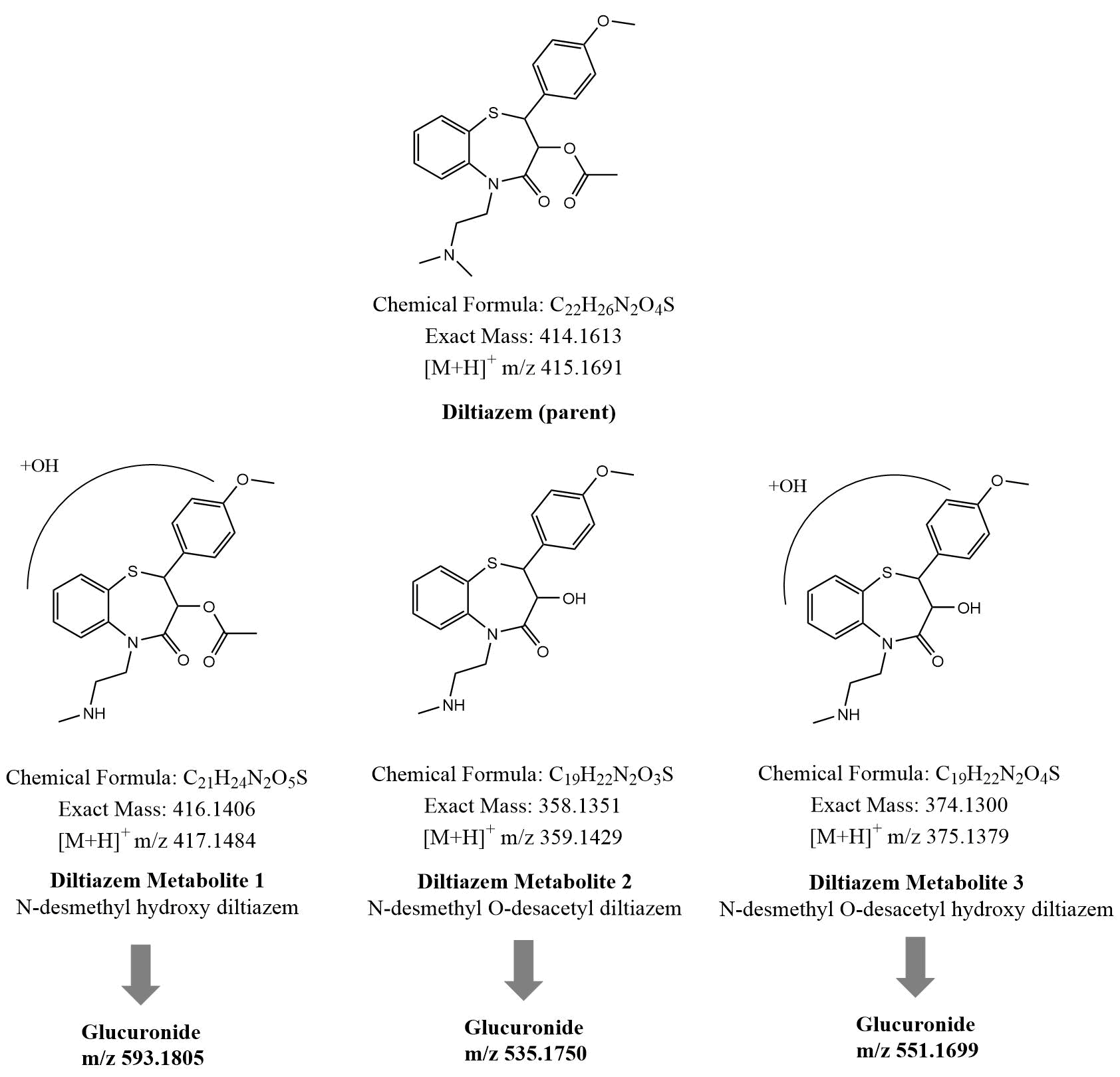


**Figure S6.** **Proposed structures of three diltiazem Phase 1 metabolites that were detected as glucuronide forms.**

## Unknown nucleoside with adenosyl moiety

The examination of the MS spectra allowed determination of the feature ion type as [M+H]+ and the corresponding molecular formula of C10H11N5O3.

Tandem mass spectrometry with collision induced dissociation (MS/MS) yielded two fragments at *m/z* 136.06 and *m/z* 97.03 (Figure S7, part A), similar to the fragmentation pattern of S-adenosylmethionine eluting at the later retention time of 6.3 min in the UHPOS assay (Figure S7, part B). This information suggests the presence of adenosyl moiety in the structure of the unknown feature. A search for possible structures for this molecular formula yielded several isomers of anhydroadenosine. However, confirmation of this possible annotation using authentic reference materials was not possible at this time.

An analysis of dataset-wide intensity correlation between the feature in question and all features in UHPOS dataset yielded high values (pearson, r>0.8) for two features corresponding to 5-methylcytidine (Table 5 and Table S4).

The MS/MS fragmentation pattern together with the correlation data lead to the annotation of the unknown feature as an “unknown nucleoside with adenosyl moiety”.


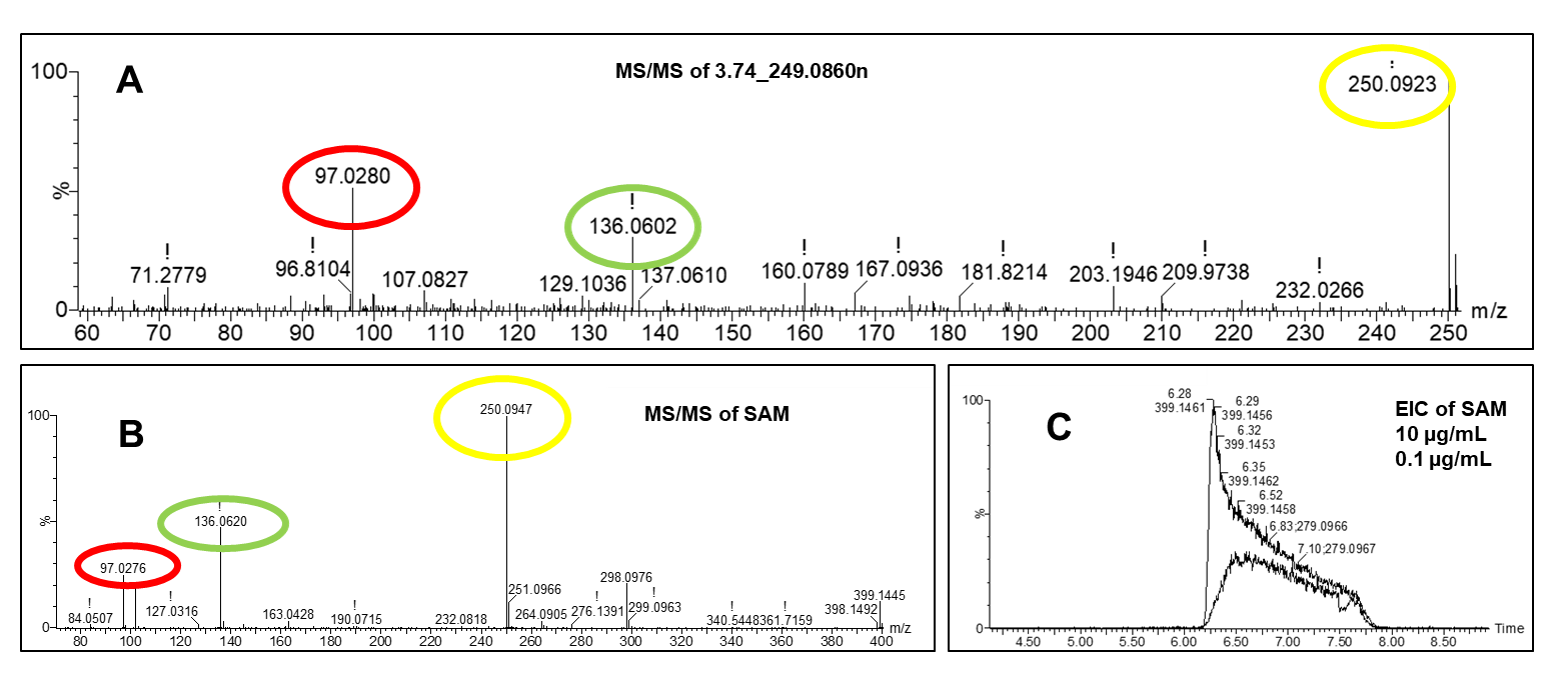


**Figure S7.** **Comparison of MS/MS spectra of the metabolite annotated as “unknown nucleoside with adenosyl moiety” detected in UHPOS assay with a retention time of 3.74 min and at m/z value of 250.093.** **(A)** MS/MS of the detected metabolic feature from UHPOS assay at 3.74 min and m/z 250.093; **(B)** MS/MS of S-adenosylmethionine (SAM) showing two fragments at m/z 136.06 and m/z 97.03; **(C)** extracted ion chromatogram (EIC) of SAM at two spiked in concentrations of 0.1 and 10 µg/mL eluting at 6.3 min.
