# Supplementary material for: Urinary metabolic phenotyping for Alzheimer’s disease
Source: Sci Rep. 2020 Dec 10;10:21745. doi: 10.1038/s41598-020-78031-9 (PMC7730184; doi:10.1038/s41598-020-78031-9)
Supplement: Supplementary file 1 — Supplementary Information. [file 41598_2020_78031_MOESM1_ESM.zip › SupplementaryData/SupplementaryFigures.docx]

**Urinary metabolic phenotyping for Alzheimer’s disease**

## Supplementary Figures


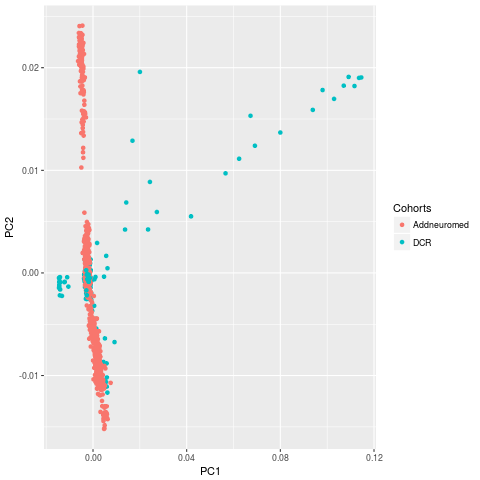


**Figure S1. Population stratification by cohort.** Principal component analysis (PCA) scatterplot illustrating the population stratification of genomics data by cohort.


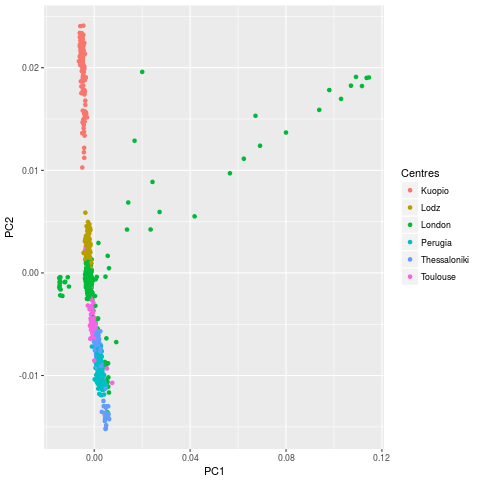


**Figure S2.** **Population stratification by samples collection centres.** Principal component analysis (PCA) scatterplot illustrating the population stratification of genomics data by sample collection centres.


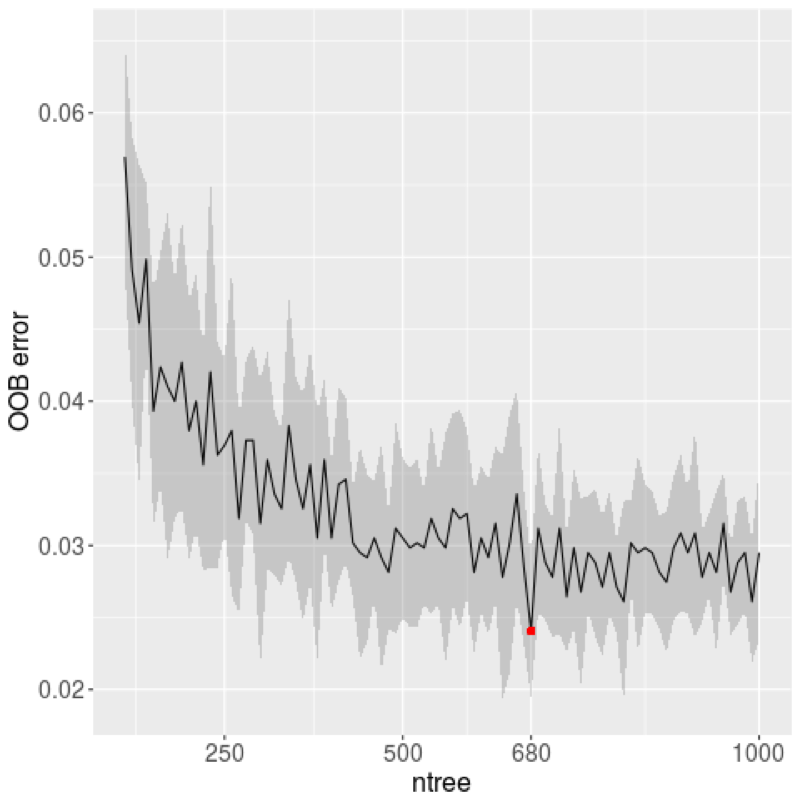


**Figure S3.** **Random Forest algorithm, tuning of “ntree” parameter**. The OOB error rates for the final mode when the number of trees in the forest (ntree) is gradually increased from 1 to 1000. The mean values of OOB error rates after 10 iterations for every “ntree” parameter value are shown as black lines with grey margins indicating standard deviations.


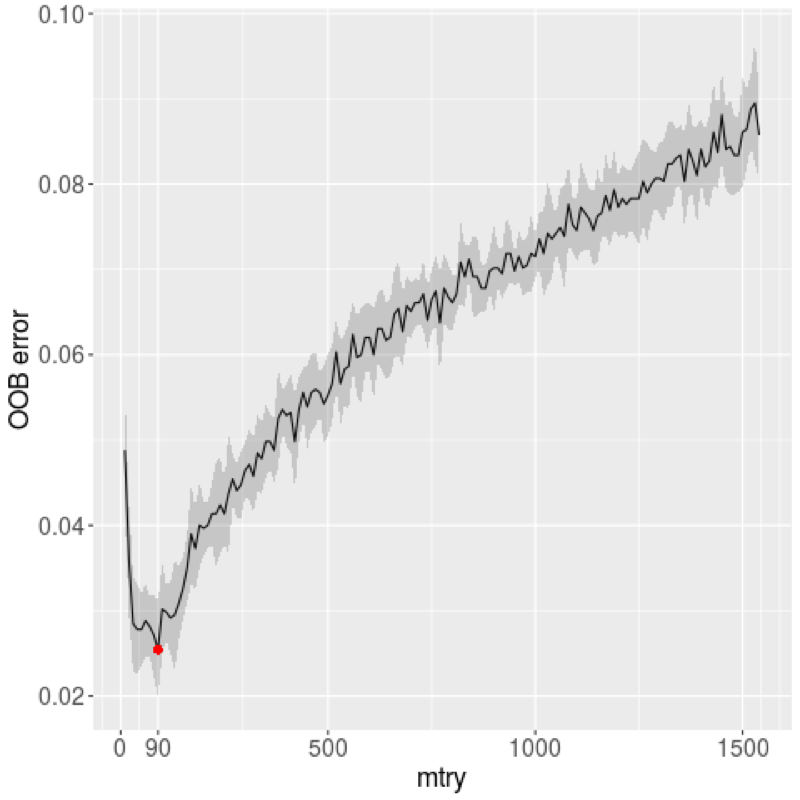


**Figure S4.** **Random Forest algorithm, tuning of “mtry” parameter**. The OOB error rates for the final model when the number of variables per split (mtry) is increased from 1 to 1545. The mean values of OOB error rates after 10 iterations for every “mtry” parameter value are shown as black lines with grey margins indicating standard deviations.
