# Supplementary material for: Urinary metabolic phenotyping for Alzheimer’s disease
Source: Sci Rep. 2020 Dec 10;10:21745. doi: 10.1038/s41598-020-78031-9 (PMC7730184; doi:10.1038/s41598-020-78031-9)
Supplement: Supplementary file 1 — Supplementary Information. [file 41598_2020_78031_MOESM1_ESM.zip › SupplementaryData/Title_page.docx]

**Urinary metabolic phenotyping for Alzheimer’s disease**

Natalja Kurbatova^1*^, Manik Garg^1^, Luke Whiley^2,3^, Elena Chekmeneva^2^, Beatriz Jiménez^2^, Maria Gomez Romero^2^, Jake Pearce^2^, Torben Kimhofer^4^, Ellie D’Hondt^5^, Hilkka Soininen^6,11^, Iwona Kłoszewska^7,11^, Patrizia Mecocci^8,11^, Magda Tsolaki^9,11^, Bruno Vellas^10,11^, Dag Aarsland ^11,12^, Alejo Nevado-Holgado^18^, Benjamine Liu ^18^, Stuart Snowden^12^, Petroula Proitsi^12^, Nicholas J. Ashton^12,15,16,17^, Abdul Hye^12^, Cristina Legido-Quigley^12^, Matthew R. Lewis^2^, Jeremy K Nicholson^2,13^, Elaine Holmes^3,13,14^, Alvis Brazma^1^, Simon Lovestone ^11, 18, 19^

* Correspondence to [natalja@ebi.ac.uk](mailto:natalja@ebi.ac.uk)

^1^ European Molecular Biology Laboratory, European Bioinformatics Institute, EMBL-EBI, Wellcome Trust Genome Campus, Hinxton, CB10 1SD, UK

^2^ MRC-NIHR National Phenome Centre, Imperial College London, Hammersmith Hospital, London W12 0NN, UK

^3^ UK Dementia Research Institute, Imperial College London, Hammersmith Hospital, London, W12 0NN, UK

^4^ Division of Systems Medicine, Imperial College London, South Kensington Campus, London SW7 2AZ, UK

^5^ IMEC, Leuven, Belgium

^6^ Department of Neurology, University of Eastern Finland and Kuopio University Hospital, Kuopio, Finland.

^7^ Medical University of Lodz, Lodz, Poland.

^8^ Institute of Gerontology and Geriatrics, University of Perugia, Perugia, Italy.

^9^ 3rd Department of Neurology, Aristotle University, Thessaloniki, Greece.

^10^ INSERM U 558, University of Toulouse, Toulouse, France

^11^ on behalf of AddNeuroMed consortium

^12^ King’s College London, Institute of Psychiatry, Psychology and Neuroscience, London, UK

^13^ Present address: Health Futures Institute, Murdoch University, Perth WA 6105, Australia

^14^ Present address: The Perron Institute for Neurological and Translational Science, Nedlands, WA 6009, Australia

^15^ Department of Psychiatry and Neurichemistry, Institute of Neuroscience and Physiology, The Sahlgrenska Academy, University of Gothenburg, Sweden

^16^ Wallenberg Centre for Molecular and Translation Medicine, University of Gothenburg, Sweden

^17^ NIHR Biomedical Research Centre of Mental Health and Biomedical Research Unit for Dementia at South London and Maudsley NHS Foundation, London, UK

^18^ Department of Psychiatry, Warneford Hospital, University of Oxford, Oxford, UK

^19^ Currently an employee of Janssen-Cilag Ltd, UK
