# Supplementary figures and images for: Urinary metabolic phenotyping for Alzheimer’s disease
Source: Sci Rep. 2020 Dec 10;10:21745. doi: 10.1038/s41598-020-78031-9 (PMC7730184; doi:10.1038/s41598-020-78031-9)

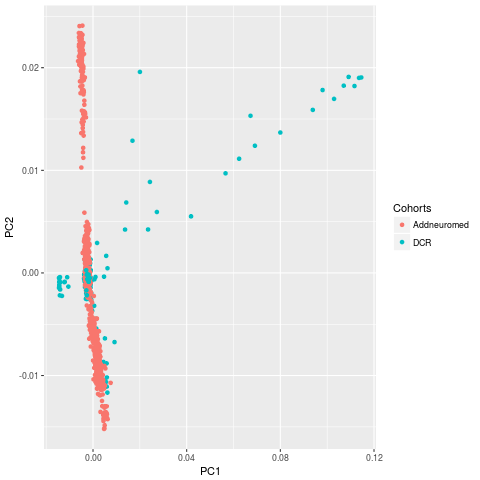

Supplement: Supplementary file 1 — Supplementary Information. [file 41598_2020_78031_MOESM1_ESM.zip › SupplementaryData/FigureS1_population_startification_cohorts.png]

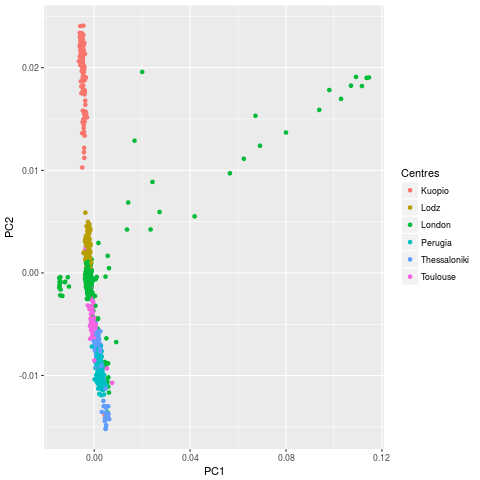

Supplement: Supplementary file 1 — Supplementary Information. [file 41598_2020_78031_MOESM1_ESM.zip › SupplementaryData/FigureS2_population_startification_centres_DCR_and_Addneuromed.png]

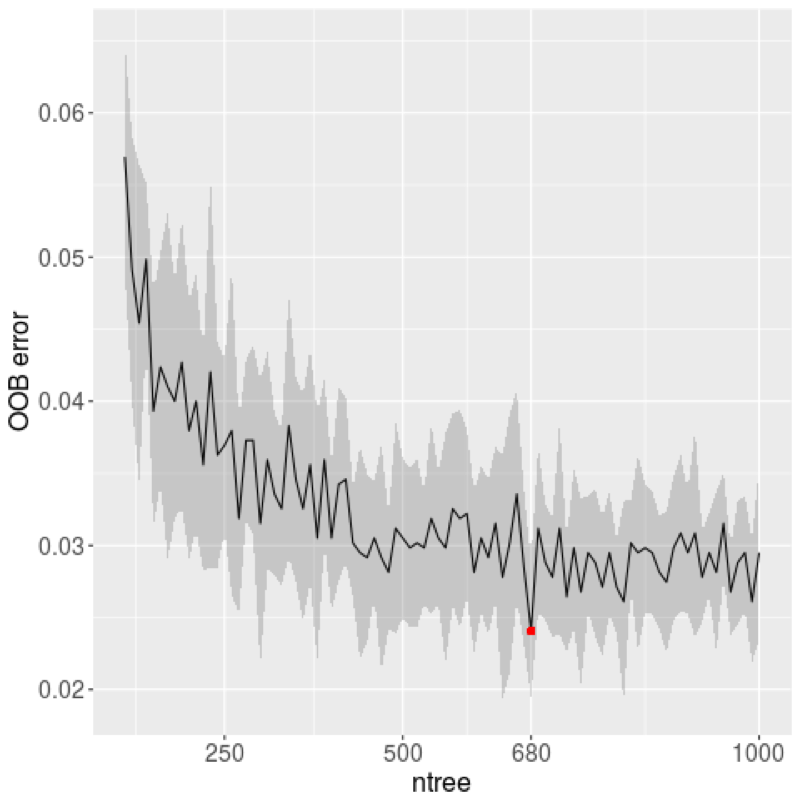

Supplement: Supplementary file 1 — Supplementary Information. [file 41598_2020_78031_MOESM1_ESM.zip › SupplementaryData/FigureS3_RF_tuning_of_ntree.png]

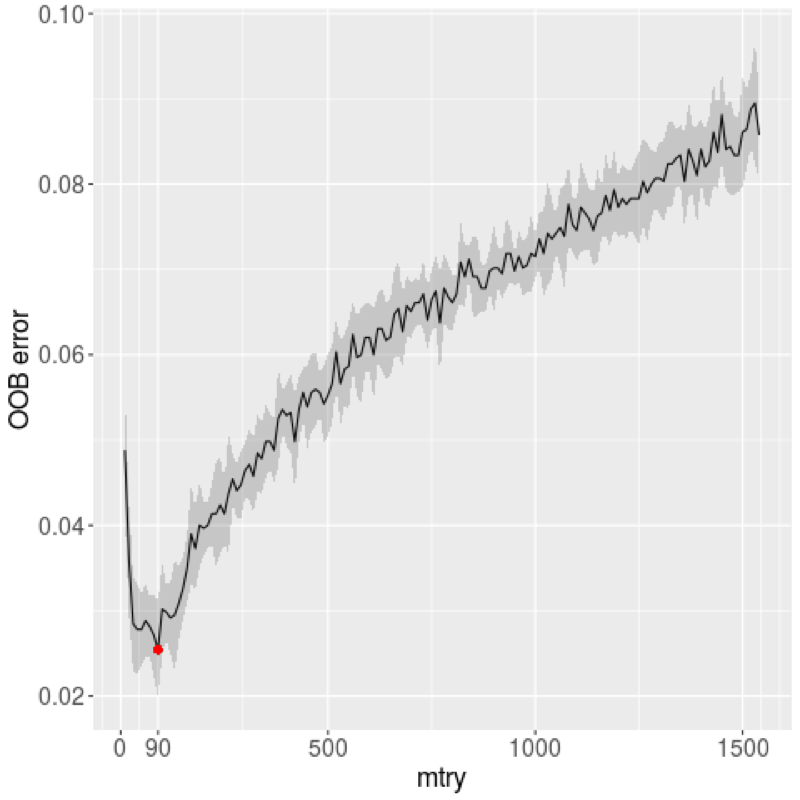

Supplement: Supplementary file 1 — Supplementary Information. [file 41598_2020_78031_MOESM1_ESM.zip › SupplementaryData/FigureS4_RF_tuning_of_mtry.png]

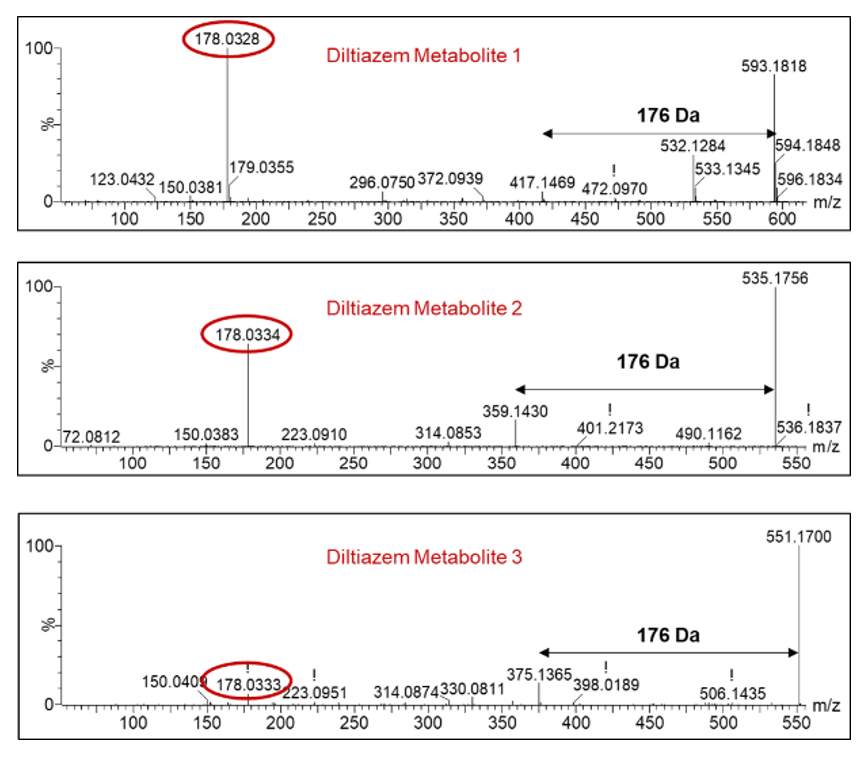

Supplement: Supplementary file 1 — Supplementary Information. [file 41598_2020_78031_MOESM1_ESM.zip › SupplementaryData/FigureS5_diltiazem_MS_spectra.png]

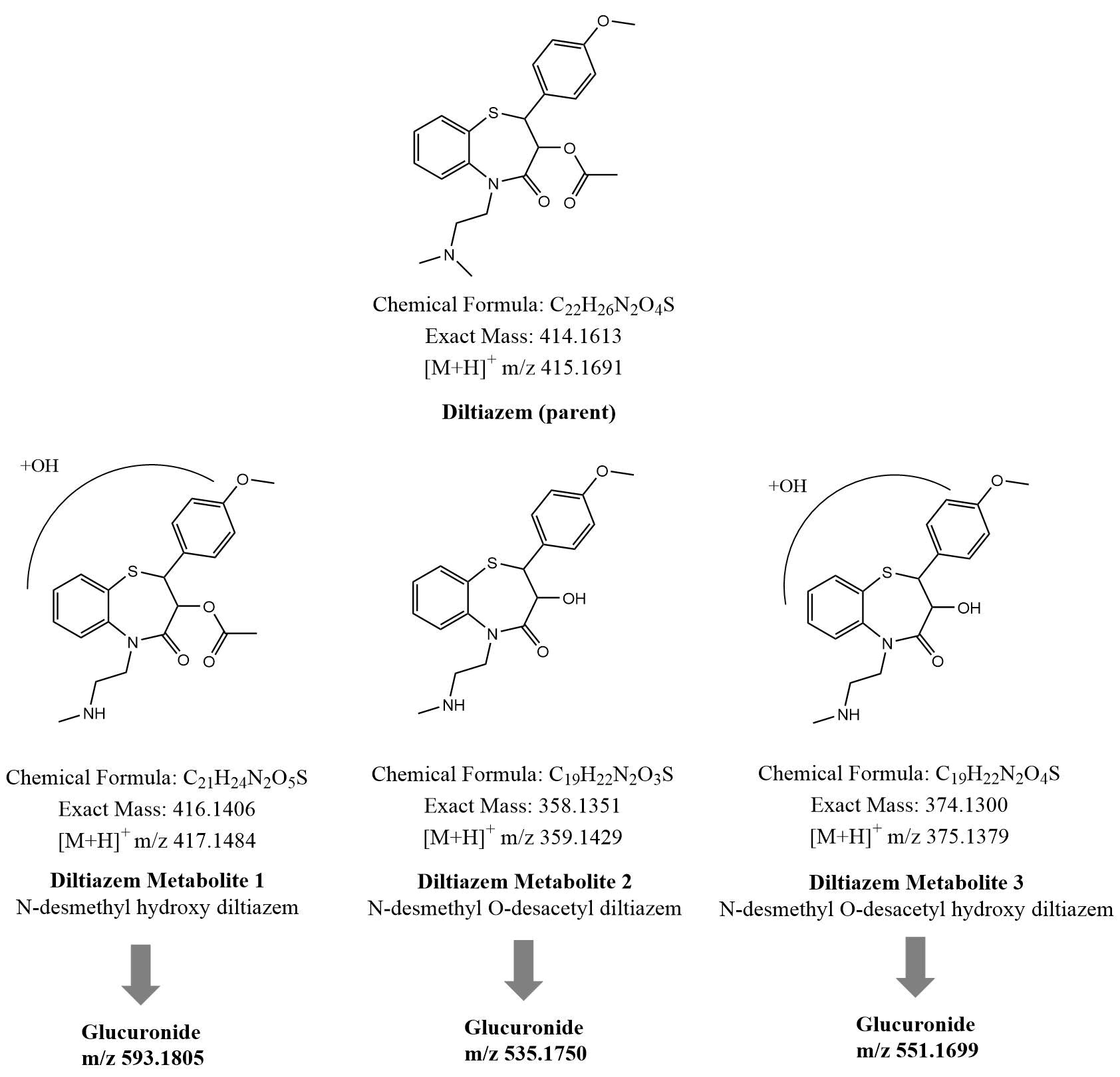

Supplement: Supplementary file 1 — Supplementary Information. [file 41598_2020_78031_MOESM1_ESM.zip › SupplementaryData/FigureS6_diltiazem_proposed_structures.png]

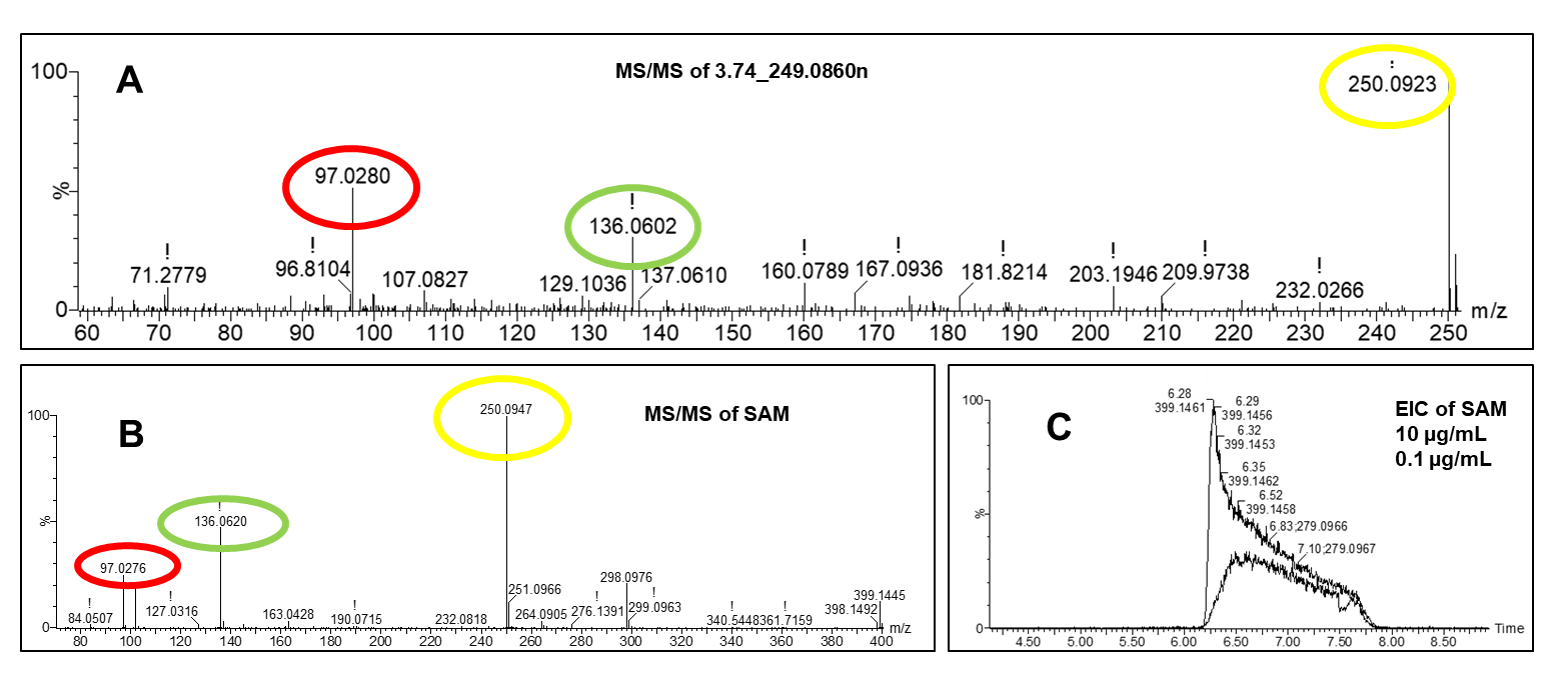

Supplement: Supplementary file 1 — Supplementary Information. [file 41598_2020_78031_MOESM1_ESM.zip › SupplementaryData/FigureS7_Unknown_nucleoside_with_adenosyl_moiety_MS_spectra.png]
